# Physical Activity and Constipation in Hong Kong Adolescents

**DOI:** 10.1371/journal.pone.0090193

**Published:** 2014-02-28

**Authors:** Rong Huang, Sai-Yin Ho, Wing-Sze Lo, Tai-Hing Lam

**Affiliations:** School of Public Health, The University of Hong Kong, Hong Kong, China; The University of Hong Kong, Hong Kong

## Abstract

**Objective:**

To examine the association of constipation with exercise, non-exercise physical activity, and sedentary behaviours in Hong Kong adolescents.

**Methods:**

In 2006–2007, 42 secondary schools were randomly selected to participate in the Hong Kong Student Obesity Surveillance (HKSOS) project. A total of 33692 Form 1–7 students (44.9% boys; mean age 14.8, SD 1.9 years) completed an anonymous questionnaire on lifestyle behaviours. Constipation was defined as a frequency of evacuation of less than once every two days. Exercise (moderate-to-vigorous levels) and non-exercise physical activity (NEPA) were each considered insufficient when less than 1 hour per day, and sedentary behaviours were considered excessive when over 4 hours per day. Logistic regression was used to calculate adjusted odds ratio (AOR) for constipation in relation to exercise, NEPA, and sedentary behaviours, adjusting for potential confounders.

**Results:**

Constipation was identified in 15.6% (95% CI 15.2% – 16.0%) of adolescents overall, 14.0% in those with sufficient exercise and 19.6% in those without. Constipation was associated with insufficient exercise (AOR 1.26, 95% CI 1.16 – 1.36), insufficient NEPA 1.21 (1.10 – 1.33) and excessive sedentary behaviours (1.25, 1.17 – 1.34). Compared with having none of the above 3 inactive behaviours, increasing AORs of constipation were observed for having 1 (AOR 1.23), 2 (AOR 1.57) and 3 (AOR 1.88) inactive behaviours (p for trend <0.001).

**Conclusions:**

Constipation was associated with insufficient physical activity and excessive sedentary behaviours among Chinese adolescents with a dose-response relation. If the association is causal, constipation could be prevented by promotion of physical activity.

## Introduction

Constipation is common worldwide affecting all ages with prevalence rates of 0.7%–29.6% in children and adolescents.[1] Physical and emotional well-being is affected and treatment could be costly.[2], [3] Previously identified risk factors of constipation include female sex, older age, poor socioeconomic status, insufficient fruit and vegetable consumption, psychological problems and insufficient physical activity.[4]–[8]

Physical activity can be classified into exercise and non-exercise physical activity (NEPA). Exercise, which generally refers to structured and planned activities of moderate-to-vigorous level, such as jogging, brisk walking and water aerobics, was negatively associated with constipation in adults.[9], [10] Daily moderate exercise was associated with a 44% reduction in risk of constipation in women.(10) Regular exercise has been recommended in elderly constipated patients,[2], [11], [12] although the causal role of diminished exercise leading to constipation has not been confirmed.[4] However, studies focusing on exercise and constipation in children and adolescents were few and results inconsistent. Two studies on children have reported a negative association between physical activity and constipation, [8], [13] while another study on adolescents found no association between moderate-to-vigorous exercise and constipation.[14]

NEPA usually refers to mild level unstructured activity, such as walking to school/work and stair climbing with energy requirement between sleeping and sports-like exercise. NEPA is an important component of energy expenditure because of its longer duration than exercise.[15] Several studies showed that walking was positively associated with bowel movement.[16], [17] One study reported that in the preparation for colonoscopy, patients who did walking exercise had better colon cleansing than those who rested.[16] Similarly, another study found walking less than 0.5 km per day was associated with an increased risk of constipation in elderly people.[17] The association between NEPA and constipation in children remains unknown.

Children and adolescents were recommended to have at least 60 minutes of moderate-to-vigorous physical activity daily.[18] Physical activity below this recommendation is considered insufficient. Television viewing is a common sedentary behaviour taking up an average of 2.5–3 hours of adolescents' leisure time per day, and computer use is also prevalent with the increasing popularity of Internet access, taking up 1.5–2 hours.[19] Screen time of more than 4 hours per day is considered excessive.[20] Insufficient physical activity and sedentary behaviours often coexist in adolescents in modern society, however, only one study, on Taiwanese adolescents, has examined the association between excessive sedentary behaviour and constipation, and found no significant association.[14]

As chronic constipation is often neglected when it first occurred while young, [21] understanding the risk factors of constipation in adolescents is particularly important. Therefore, we examined the association of constipation with insufficient physical activity, NEPA, and sedentary behaviours (television/video viewing and network use/computer game playing) in Hong Kong adolescents.

## Methods

### Ethics statements

Parents were informed of the survey through an invitation letter and agreement for their children to participate was assumed unless they returned a reply slip included in the letter. Even with parental agreement, student participation was voluntary. Ethical approval was granted by the Institutional Review Board of the University of Hong Kong/Hospital Authority Hong Kong, West Cluster, including the passive consent procedure with written consent waived.

### Sampling and Subjects

In the Hong Kong Student Obesity Surveillance (HKSOS) project 2006-07, 42 schools were randomly selected to represent all mainstream non-international secondary schools in Hong Kong in school district, source of funding, language of instruction (Chinese/English), religious background (Christian, others, none) and school type (co-education or single sex). More details on sampling can be found elsewhere.[22]–[24] A total of 33692 students aged 11–18 (84.5% response rate)[22] completed an anonymous questionnaire on obesity and lifestyle in classroom. Non-respondents were either absent or those who refused to participate. Regression analyses were based on 26864 (79.7% of 33692) students, excluding 6828 students with missing data in socio-demographic characteristics or constipation status. Excluded and included students were comparable in sex, age, highest parental education, and perceived family affluence (Cohen's d from 0.08 to 0.26, table not shown).

### Measures

#### Constipation

Students were asked “How often do you have a bowel movement” with 5 response options of once every three days or less frequently, once every two days, once every day, twice every day, and three times every day or more frequently. Constipation was defined as once every three days or less frequently consistent with one of the symptoms in Rome III criteria of “fewer than three defecations per week” and the definition used in previous studies.[2], [10], [25], [26]

#### Physical activity and sedentary behaviours

The variables of exercise and non-exercise physical activity have also been used in our previous paper.[23] Students were asked “How long do you usually do the following after school/during holiday (weekend day)?” in 2 separate items: (a)exercise, (b) non-exercise physical activity (NEPA), (c) TV/video viewing, and (d) Internet use/computer games. Ten response options from 30 minutes or less to 5 hours or longer were provided. Exercise referred to moderate or vigorous activities, such as running, swimming/water sports, hiking, gym/aerobic dance, whereas NEPA referred to low intensity activities embedded in daily living, such as walking to work and climbing stairs.[23] Duration spent on exercise per day was the weighted average of school days and weekend days (5:2). Based on the recommendation of at least 60 minutes of physical activity per day,[18] those who chose “one hour” or longer durations were considered having sufficient exercise (reference group) and otherwise as insufficient exercise. Without a clear standard of sufficient NEPA, the same operational definition for exercise was applied to NEPA. According to recommendations in children for not exceeding 2 hours of television/video viewing and 2 hours of Internet use/computer games per day,[27] these two behaviours were combined to calculate a weighted average of sedentary behaviours per day with a cutoff of 4 hours. A composite score from 0 to 3 was generated for the number of unhealthy behaviours of (1) insufficient exercise, (2) insufficient NEPA, and (3) excessive screen time.

#### Covariates

Students reported their socio-demographic characteristics, such as sex, age, and perceived family affluence (relatively poor/medium/relatively wealthy). Fruit and vegetable intake were assessed by asking students “On average, how many servings of (a) fruit (b) vegetable do you consume every day?” with 9 response options from none to 4 or more. Students also reported whether they felt (a) depressed and (b) anxious in the past 30 days.

#### Statistical analysis

Bivariate analyses were conducted to examine the association between student characteristics and constipation using Chi-square tests for categorical variables and Student's t-test for continuous variables. Logistic regression was used to examine the association of constipation (outcome variable) with exercise, non-exercise physical activity, and sedentary behaviours (independent variable) adjusting for each other, sex, age, perceived family affluence, fruit and vegetable intake, feeling depressed, and feeling anxious. Subsequently, logistic regression was used to examine the association between the composite score variable and constipation adjusting for the abovementioned covariates. Both crude and adjusted odds ratios and corresponding 95% CI were presented. Because irritable bowel syndrome patients may also have constipation, all the above analyses were repeated excluding 416 subjects who reported irritable bowel syndrome diagnosed by medical practitioners. Sensitivity analyses gave similar results.

## Results

Of 33692 students, 1321 (4%) were excluded due to missing frequency of bowel movement, leaving 32371 for analyses. Of these, 44.4% were boys with a mean age of 14.4 years (SD 1.9). Overall, 15.4% (95% CI 14.9–15.8) of students had constipation. Constipation was consistently more common in inactive students by physical activity (19.6% vs 14.0%, p<0.001), NEPA (19.7% vs 14.7%, p<0.001), and sedentary behaviour (17.4% vs 14.2%, p<0.001). The prevalence of constipation increased with the number of inactive behaviours from none (12.5%), one (15.5%), two (20.7%) to three (23.8%). Constipation was also associated with being female, poorer family affluence, feeling depressed and feeling anxious (Table 1).

**Table 1:** Background information of subjects with constipation and without constipation (N = 32371).

Category	Subcategory	Constipated (total = 5052)	Nonconstipated (total = 27319)	Prevalence of constipation (%)	P value
Sex	Girl	3181 (63.4)	14681 (54.2)	17.8	<0.001
	Boy	1835 (36.6)	12415 (45.8)	12.9	
Age (mean±SD yrs)	NA	5052 (14.3±1.8)	27319 (14.4±1.9)	NA	<0.001^a^
Perceived family affluence	Relatively poor	2146 (42.8)	9670 (35.6)	18.2	<0.001
	Medium	2439 (48.6)	14154 (52.1)	14.7	
	Relatively wealthy	430 (8.6)	3330 (12.3)	11.4	
Physical activity	> = 1 hr/day	3058 (65.4)	18787 (74.0)	14.0	<0.001
	<1 hr/day	1617 (34.6)	6615 (26.0)	19.6	
NEPA	> = 1 hr/day	3553 (80.9	20578 (85.8)	14.7	<0.001
	<1 hr/day	837 (19.1)	3421 (14.2)	19.7	
bSedentary behaviours	< = 4 hr/day	2543 (54.9)	15320 (60.7)	14.2	<0.001
	>4 hr/day	2086 (45.1)	9918 (39.3)	17.4	
cNumber of unhealthy behaviours	0	1419 (33.4)	9895 (42.4)	12.5	<0.001
	1	1704 (40.1)	9282 (39.7)	15.5	
	2	888 (20.9)	3407 (14.6)	20.7	
	3	243 (5.7)	780 (3.3)	23.8	
Feeling depressed	No	4835 (95.8)	26345 (96.6)	15.5	<0.001
	Yes	212 (4.2)	936 (3.4)	18.5	
Feeling anxious	No	4674 (92.6)	25682 (94.1)	15.4	<0.001
	Yes	373 (7.4)	1599 (5.9)	18.9	

a P value was from Student's t-test.

b including TV/video watching and network use/computer games.

Abbreviations: NEPA (non-exercise physical activity), NA (Not applicable).

c the sum of students in each category for each variable may not be equal to the total number due to missing values.

Table 2 shows after adjusting for covariates, insufficient exercise (AOR 1.26, 95% CI 1.16–1.36), insufficient NEPA (1.21, 1.10–1.33), TV/video viewing and Internet use/computer games (1.25, 1.17–1.34) was still associated with higher odds of constipation. Having all the three unhealthy behaviours was most strongly associated with higher odds of constipation (2.17, 1.86–2.54), while the odds ratio for having two or one unhealthy behaviour was 1.82 (1.66–1.99) and 1.28 (1.19–1.38) respectively. Logistic regression analyses were repeated in adolescents excluding 416 adolescents who self-reported irritable bowel disorder, and these sensitivity analyses gave similar results.

**Table 2:** Multivariate analyses of physical activity and sedentary behaviours and constipation.

Category	Subcategory	OR (95% CI)	OR (95%CI)
		Unadjusted	#Adjusted
Exercise	> = 1 hr/day	1	1
	<1 hr/day	1.50** (1.41–1.60)	1.26** (1.16–1.36)
NEPA	> = 1 hr/day	1	1
	<1 hr/day	1.42** (1.30–1.54)	1.21** (1.10–1.33)
Sedentary behaviours	< = 4 hrs/day	1	1
	>4 hrs/day	1.27** (1.19–1.35)	1.25** (1.17–1.34)
Number of unhealthy behaviours	0	1	1
	1	1.28** (1.19–1.38)	1.23** (1.14–1.33)
	2	1.82** (1.66–1.99)	1.57** (1.42–1.72)
	3	2.17** (1.86–2.54)	1.88** (1.60–2.20)
P value for trend		<0.001	<0.001

# adjusting for sex, age, perceived family affluence, fruit and vegetable intake, and depression and anxiety symptoms;

*p<0.05, **p<0.01.

Index: created after combining the three unhealthy behaviours (physical activity less than 1 hour/d, NEPA less than 1 hour/d, and screen time of at least 4 hours/d).

## Discussion

We found constipation was common, affecting 15% of adolescents. A slightly lower prevalence of 12% in Hong Kong children aged 6–15 was reported by Tam et al.;[28] however, direct comparison is difficult due to different age of subjects and definitions of constipation. Using the same definition of constipation, another study reported a lower prevalence of 9.3% among Taiwanese adolescents.

Constipation may be understood differently as a symptom or disease.[14] In epidemiological studies, ROME criteria, patients' self-report of straining and difficulty in passing stool, and infrequent bowel movement are commonly used to define constipation,[10], [28]–[30] while in clinical settings, the cutoff of less than three bowel movements per week is often used.[12] An acceptable agreement was found between self-report prevalence of constipation and that using ROME I and II criteria.[31] As adolescents may feel uncomfortable to discuss bowel movements and may neglect constipation, more active enquiries by health care professionals are needed.

The present study is one of the first few studies to examine the association between physical activity and constipation in adolescents.[14] The present study shows insufficient moderate-to-vigorous exercise, insufficient NEPA, and excessive sedentary behaviours were independently associated with constipation in adolescents. Our results are inconsistent with a study on Taiwanese adolescents (grades7–12 students) which showed that the time spent on sedentary behaviours was not associated with constipation defined by <3 bowel movements per week with an adjusted odds ratio of 1.0.[14] However, our results are consistent with a study on preschool children that physical activity below the recommended 60 min/day was associated with constipation.[8] Studies focused on the general population and elderly patients have also shown that immobility or insufficient physical activity was linked to constipation.[10], [32], [33] In a study of more than 60000 women, those who did moderate-to-vigorous physical activity daily was 44% less likely to report constipation (defined as less than 3 bowel movements weekly) than those who did less than once weekly.[10] Likewise, immobility was identified as one of the prominent risk factors associated with constipation in 201 elderly patients living at home.[33] It has been reported that regular modest physical activity may relieve symptoms in mild constipation patients.[11], [34] Consistently, our study showed that insufficient physical activity was associated with a higher risk of constipation in Hong Kong adolescents. Possible mechanisms by which physical activity modulates bowel movement include reduction of colonic transit time and hormonal change during physical activity.[2] Physical activity promotes bowel movements by improving bowel activity in the large intestine.[11], [34] Nevertheless, it is difficult to quantify the physiological changes caused by physical activity, because human colonic motor activity has been insufficiently investigated.[35] In addition, physical activity may change the level of endogenous sex hormones,[36] which have been shown to regulate colonic transit time, for example, reduced progesterone level could decrease the gastrointestinal transit time.[37] The negative association between physical activity and constipation could also be partly explained by the observation that physical activity may stimulate appetite, and sufficient dietary intake leads to higher frequency of bowel movement.[38] More studies are needed to elucidate the underlying physiological and behavioural mechanisms.

In 2008, a recommendation of at least 60 minutes per day of moderate-to-vigorous physical activity daily for children was published in the US.[18] Extensive studies have shown the beneficial effects of physical activity on both physical and psychosocial health.[39] About 30% of our adolescents failed to achieve this recommendation. Our study showed adolescents with insufficient physical activity were 26% more likely to be constipated, suggesting the need to increase physical activity in adolescents. This is different from the Taiwan results that duration of moderate-to-vigorous physical activity was not associated with constipation (defined as <3 bowel movements weekly).[14]

Our study showed a statistically significant positive association between insufficient NEPA and constipation. Regular exercise is declining in industrialized countries, and NEPA has been encouraged as a compensation, because NEPA can be easily accumulated without extra facilities and time.[40], [41] Moreover, one study suggested that increasing walking and standing up in daily life may be more effective in treating constipation than regular exercise.[14] Given the limited sports facilities and space for regular exercise in Hong Kong, NEPA might be recommended to constipated adolescents.

Consistent with other studies in adults and adolescents,[14], [42] our study also shows sedentary behaviours was more common in constipated adolescents. In addition to well-known hazards of TV/video viewing and Internet use/computer games on health, such as higher risk of obesity and cardiovascular diseases,[43] our study adds to the current evidence regarding the effects of excessive screening time on bowel movement. The dose-response relation between the composite score and constipation suggested that more sedentary behaviours were associated with a higher risk of constipation in adolescents.

Our study has several limitations. First, constipation was defined based on self-reports of bowel movements instead of ROME III criteria. As infrequent bowel movement is a key symptom of ROME III criteria and is often used to define constipation by clinicians, using a single item assessing frequency is common in large-scale epidemiological studies,[10] and could facilitate responses from subjects. Further studies measuring other aspects of defecation are warranted, such as the shape of stool, and abdominal pain or discomfort during defecation, to more accurately examine the association between physical activity and constipation symptoms in adolescents. Second, physical activity and screening time were not objectively measured. However, findings based on the same database that male sex, younger age and holidays were positively associated with physical activity and NEPA have supported the validity of these self-reported activities.[23] Third, 27% of adolescents with incomplete data were excluded; however, the socio-demographic characteristics of those excluded and included were similar. Moreover, missing data were mostly due to physical activity and NEPA (21% missing rate), but not constipation. This suggests that non-response is unlikely to be a major source of confounding and bias. Fourth, temporal relation between insufficient physical activity and constipation cannot be ascertained using cross-sectional data. Nevertheless, constipation may lead to an increase in physical activity as a remedy or decrease in physical activity due to discomfort, making reverse causality an unlikely explanation of the observed associations. Finally, residual confounding cannot be ruled out. Simple measures of daily fruit and vegetable consumption could not accurately reflect fiber intake, which may protect against constipation.[10] Additionally, we have not collected information on concomitant diseases and medication use that may impact both physical activity and constipation, although these conditions were unlikely to be common in adolescents. Furthermore, any use of medication to treat constipation in those who were inactive would have resulted in an underestimation of the association between physical inactivity and constipation.

## Conclusions

Constipation was common in Hong Kong adolescents. Insufficient physical activity and excessive sedentary behaviours were positively associated with constipation with dose-response relation. If the association is causal, promotion of physical activity and reduction in sedentary behaviours may help relieve constipation in adolescents. Parents and primary care physicians may help adolescents identify constipation and advise on lifestyle changes including an increase in physical activity.

## References

[1] Mugie SM, Di Lorenzo C, Benninga MA (2011) Constipation in childhood. Nature reviews Gastroenterology & hepatology 8: 502–11. 21808283 10.1038/nrgastro.2011.130

[2] Mugie SM, Benninga MA, Di Lorenzo C (2011) Epidemiology of constipation in children and adults: a systematic review. Best practice & research Clinical gastroenterology 25: 3–18. 21382575 10.1016/j.bpg.2010.12.010

[3] Youssef NN, Langseder AL, Verga BJ, Mones RL, Rosh JR (2005) Chronic childhood constipation is associated with impaired quality of life: A case-controlled study. Journal of pediatric gastroenterology and nutrition 41: 56–60. 15990631 10.1097/01.mpg.0000167500.34236.6a

[4] Locke GR, Pemberton JH, Phillips SF (2000) AGA technical review on constipation. American Gastroenterological Association. Gastroenterology 119: 1766–78. 11113099 10.1053/gast.2000.20392

[5] Suares NC, Ford AC (2011) Prevalence of, and risk factors for, chronic idiopathic constipation in the community: systematic review and meta-analysis. American Journal of Gastroenterology 106: 1582–91. 21606976 10.1038/ajg.2011.164

[6] Cheng C, Chan AOO, Hui WM, Lam SK (2003) Coping strategies, illness perception, anxiety and depression of patients with idiopathic constipation: a population-based study. Aliment Pharm Therap 18: 319–26. 10.1046/j.1365-2036.2003.01663.x 12895216

[7] Lee WTK, Ip KS, Chan JSH, Lui NW, Young BW (2008) Increased prevalence of constipation in pre-school children is attributable to under-consumption of plant foods: A community-based study. Journal of paediatrics and child health 44: 170–5. 17854410 10.1111/j.1440-1754.2007.01212.x

[8] Driessen LM, Jong J, Wijtzes A, de Vries SI, Jaddoe VW, et al (2013) Physical activity and functional constipation during the preschool period: the generation R study. Journal of pediatric gastroenterology and nutrition 57: 768–74. 23857342 10.1097/MPG.0b013e3182a313fc

[9] Everhart JE, Go VL, Johannes RS, Fitzsimmons SC, Roth HP, et al (1989) A longitudinal survey of self-reported bowel habits in the United States. Digestive diseases and sciences 34: 1153–62. 2787735 10.1007/BF01537261

[10] Dukas L, Willett WC, Giovannucci EL (2003) Association between physical activity, fiber intake, and other lifestyle variables and constipation in a study of women. American Journal of Gastroenterology 98: 1790–6. 12907334 10.1111/j.1572-0241.2003.07591.x

[11] Muller-Lissner SA, Kamm MA, Scarpignato C, Wald A (2005) Myths and misconceptions about chronic constipation. American Journal of Gastroenterology 100: 232–42. 15654804 10.1111/j.1572-0241.2005.40885.x

[12] Hsieh C (2005) Treatment of constipation in older adults. American family physician 72: 2277–84. 16342852

[13] Inan M, Aydiner CY, Tokuc B, Aksu B, Ayvaz S, et al (2007) Factors associated with childhood constipation. Journal of paediatrics and child health 43: 700–6. 17640287 10.1111/j.1440-1754.2007.01165.x

[14] Chien L-Y, Liou YM, Chang P (2011) Low defaecation frequency in Taiwanese adolescents: Association with dietary intake, physical activity and sedentary behaviour. Journal of paediatrics and child health 47: 381–6. 21309885 10.1111/j.1440-1754.2010.01990.x

[15] Levine JA (2002) Non-exercise activity thermogenesis (NEAT). Best Pract Res Cl En 16: 679–702. 10.1053/beem.2002.0227 12468415

[16] Kim HS, Park DH, Kim JW, Jee MG, Baik SK, et al (2005) Effectiveness of walking exercise as a bowel preparation for colonoscopy: a randomized controlled trial. The American journal of gastroenterology 100: 1964–9. 16128940 10.1111/j.1572-0241.2005.40373.x

[17] Kinnunen O (1991) Study of constipation in a geriatric hospital, day hospital, old people's home and at home. Aging (Milan, Italy) 3: 161–70. 10.1007/BF03323997 1911905

[18] Are these special recommendations for young people. Centers for Disease Control and Prevention, US. Available: http://www.cdc.gov/physicalactivity/. Accessed 2013 Oct 3.

[19] Nelson MC, Neumark-Stzainer D, Hannan PJ, Sirard JR, Story M (2006) Longitudinal and secular trends in physical activity and sedentary behavior during adolescence. Pediatrics 118: e1627–e34. 17142492 10.1542/peds.2006-0926

[20] Carson V, Janssen I (2011) Volume, patterns, and types of sedentary behavior and cardio-metabolic health in children and adolescents: a cross-sectional study. BMC Public Health 11: 274. 21542910 10.1186/1471-2458-11-274 PMC3112118

[21] Khan S, Campo J, Bridge JA, Chiappetta LC, Wald A, et al (2007) Long-term outcome of functional childhood constipation. Digestive diseases and sciences 52: 64–9. 17151806 10.1007/s10620-006-9308-9

[22] Mak KK, Ho SY, Thomas GN, Lo WS, Cheuk DK, et al (2010) Smoking and sleep disorders in Chinese adolescents. Sleep medicine 11: 268–73. 20176504 10.1016/j.sleep.2009.07.017

[23] Mak KK, Ho SY, Lo WS, McManus AM, Lam TH (2011) Prevalence of exercise and non-exercise physical activity in Chinese adolescents. The international journal of behavioral nutrition and physical activity 8: 3. 21251295 10.1186/1479-5868-8-3 PMC3036595

[24] Lo WS, Ho SY, Mak KK, Lai YK, Lam TH (2009) Adolescents' experience of comments about their weight - prevalence, accuracy and effects on weight misperception. BMC Public Health 9: 271. 19642972 10.1186/1471-2458-9-271 PMC2731749

[25] Drossman DA, Sandler RS, Mckee DC, Lovitz AJ (1982) Bowel patterns among subjects not seeking health-care - use of a questionnaire to identify a population with bowel dysfunction. Gastroenterology 83: 529–34. 7095360

[26] Anti M, Pignataro G, Armuzzi A, Valenti A, Lascone E, et al (1998) Water supplementation enhances the effect of high-fiber diet on stool frequency and laxative consumption in adult patients with functional constipation. Hepato-Gastroenterol 45: 727–32. 9684123

[27] Bar-on ME, Broughton DD, Buttross S, Corrigan S, Gedissman A, et al (2011) Children, adolescents, and television. Pediatrics 107: 423–6.

[28] Tam YH, Li AM, So HK, Shit KY, Pang KK, et al (2012) Socioenvironmental factors associated with constipation in Hong Kong children and Rome III criteria. Journal of pediatric gastroenterology and nutrition 55: 56–61. 22197949 10.1097/MPG.0b013e31824741ce

[29] Wald A, Scarpignato C, Mueller-Lissner S, kamm MA, Hinkel U, et al (2008) A multinational survey of prevalence and patterns of laxative use among adults with self-defined constipation. Aliment Pharm Therap 28: 917–30. 10.1111/j.1365-2036.2008.03806.x 18644012

[30] Campbell AJ, Busby WJ, Horwath CC (1993) Factors associated with constipation in a community based sample of people aged 70 years and over. Journal of Epidemiology and Community Health 47: 23–6. 8382251 10.1136/jech.47.1.23 PMC1059704

[31] Garrigues V, Galvez C, Ortiz V, Ponce M, Nos P, et al (2004) Prevalence of constipation: Agreement among several criteria and evaluation of the diagnostic accuracy of qualifying symptoms and self-reported definition in a population-based survey in Spain. Am J Epidemiol 159: 520–6. 14977649 10.1093/aje/kwh072

[32] Sodergren M, McNaughton S, Salmon J, Ball K, Crawford DA, et al (2012) Associations between fruit and vegetable intake, leisure-time physical activity, sitting time and self-rated health among older adults: cross-sectional data from the WELL study. Bmc Public Health 12: 551. 22830932 10.1186/1471-2458-12-551 PMC3487826

[33] Donald I, Smith R, Cruikshank J, Elton RA, Stoddard ME (1985) A study of constipation in the elderly living at home. Gerontology 31: 12–8. 10.1159/000212689 2987086

[34] De Schryver AM, Keulemans YC, Peters HP, Akkermans LM, Smout AJ, et al (2005) Effects of regular physical activity on defecation pattern in middle-aged patients complaining of chronic constipation. Scandinavian journal of gastroenterology 40: 422–9. 16028436 10.1080/00365520510011641

[35] Bassotti G, Clementi M, Antonelli E, Pelli MA, Tonini M, et al (2001) Low-amplitude propagated contractile waves: a relevant propulsive mechanism of human colon. Digestive and Liver Disease 33: 36–40. 11303973 10.1016/s1590-8658(01)80133-x

[36] Chan M-F, Dowsett M, Folkerd E, Bingham S, Wareham N, et al (2007) Usual physical activity and endogenous sex hormones in postmenopausal women: The European prospective investigation into Cancer–Norfolk population study. Cancer Epidemiology Biomarkers & Prevention 16: 900–5. 10.1158/1055-9965.EPI-06-0745 17507613

[37] Chiarelli Pa, Brown W, McElduff P (2000) Constipation in Australian women: prevalence and associated factors. International Urogynecology Journal 11: 71–8. 10.1007/s001920050073 10805262

[38] Blundell JE, Stubbs RJ, Hughes DA, Whybrow S, king NA (2003) Cross talk between physical activity and appetite control: does physical activity stimulate appetite? Proceedings of the Nutrition Society 62: 651–61. 14692601 10.1079/PNS2003286

[39] Biddle SJ, Gorely T, Stensel DJ (2004) Health-enhancing physical activity and sedentary behaviour in children and adolescents. Journal of Sports Sciences 22: 679–701. 15370482 10.1080/02640410410001712412

[40] Eves FF, Masters RS, Mcmanus A, leung M, Wong P, et al (2008) Contextual barriers to lifestyle physical activity interventions in Hong Kong. Medicine and science in sports and exercise 40: 965. 18408599 10.1249/MSS.0b013e3181659c68

[41] Eves FF, Masters RS (2006) An uphill struggle: Effects of a point-of-choice stair climbing intervention in a non-English speaking population. International Journal of Epidemiology 35: 1286–90. 16849368 10.1093/ije/dyl141

[42] Whitehead WE, Drinkwater D, Cheskin LJ, Heller BR, Schuster MM (1989) Constipation in the elderly living at home. Definition, prevalence, and relationship to lifestyle and health status. Journal of the American Geriatrics Society 37: 423. 2539405 10.1111/j.1532-5415.1989.tb02638.x

[43] Rey-López JP, Vicente-Rodríguez G, Biosca M, Moreno LA (2008) Sedentary behaviour and obesity development in children and adolescents. Nutrition, Metabolism and Cardiovascular Diseases 18: 242–51. 10.1016/j.numecd.2007.07.008 18083016

